# The psycho-social and clinical profile of women referred for psycho-legal evaluation to forensic mental health units in South Africa

**DOI:** 10.4102/sajpsychiatry.v25i0.1230

**Published:** 2019-02-21

**Authors:** Mohammed Nagdee, Lillian Artz, Carmen Corral-Bulnes, Aisling Heath, Ugasvaree Subramaney, Helena G. de Clercq, Helmut Erlacher, Carla Kotze, Gian Lippi, Samantha Naidoo, Funeka Sokudela

**Affiliations:** 1 Fort England Hospital, South Africa; 2 Department of Psychiatry, Walter Sisulu University, South Africa; 3 Department of Psychology, Rhodes University, South Africa; 4 Gender, Health and Justice Research Unit, University of Cape Town, South Africa; 5 Sterkfontein Hospital, South Africa; 6 Department of Psychiatry, University of the Witwatersrand, South Africa; 7 Valkenberg Hospital, South Africa; 8 Department of Psychiatry, University of Cape Town, South Africa; 9 Weskoppies Hospital, South Africa; 10 Department of Psychiatry, University of Pretoria, South Africa

## Abstract

**Background:**

There is a paucity of research on women offenders in the South African context, particularly those referred for forensic psychiatric observation. Little is known about their life histories, the nature of their offences or the psycho-social contexts that enable, or are antecedents to, women’s criminal offending.

**Aims:**

This research study, the largest of its kind in South Africa, examined the psycho-social contexts within which women offenders referred for psychiatric evaluation come to commit offences. The profiles of both offenders and victims, as well as reasons for referral and forensic mental health outcomes, were investigated.

**Methods:**

A retrospective record review of 573 cases, spanning a 12-year review period, from six different forensic psychiatric units in South Africa, was conducted.

**Results:**

The findings describe a population of women offenders who come from backgrounds of socio-demographic and socio-economic adversity, with relatively high pre-offence incidences of being victims of abuse themselves, with significant levels of mental ill-health and alcohol abuse permeating their life histories. The majority of index offences which led to court-ordered forensic evaluations were for violent offences against the person, with murder being the single most common index offence in the sample. Most victims of violence were known to the accused. There were also relatively high rates of psychotic and mood-spectrum disorders present, with relatively low rates of personality disorders. The majority of women were deemed to be trial competent and criminally responsible in relation to their index offences.

**Conclusion:**

It is recommended that more standardised and gender-sensitive forensic mental health assessment approaches, documentation and reporting be employed throughout the country. Future research should compare male and female offending patterns and forensic mental health profiles.

## Background

Empirically, female offenders have received little attention in comparison with male offenders, but the few South African studies that do exist have shown that women’s pathways to imprisonment are characterised by prior sexual and physical victimisation, parental neglect, stressful life events, substance abuse and mental health issues.^1,2,3,4^ These studies also reflect findings in international research on women in prison, which has found a number of childhood, psycho-social and familial criminogenic risk factors associated with offending behaviour, including early onset impulsivity and conduct disorder, low educational and occupational attainment, adverse early life experiences, poor child-rearing practices, single parenting, large and chaotic family environments, parental (especially paternal) criminality, substance misuse and socio-economic deprivation.^5,6,7,8^ Studies from both the United Kingdom and the United States also reveal a consistent picture with respect to mental ill-health of female detainees: female prisoners are around twice as likely as their male counterparts to have a psychiatric diagnosis.^9,10,11^ Women prisoners present with particularly high rates of self-injurious behaviour, suicidal tendencies, substance misuse, personality disorders, intellectual disability, depressive and anxiety disorders, and HIV.^6,12,13,14,15^

With the exception of Offen,^16^ whose study showed indications that gender and ethnicity played a role in the process of court referrals, and more recently, Khoele et al.^17^ who specifically examined the forensic clinical records of 32 women charged with murder or attempted murder of minors referred to Weskoppies Hospital (in Pretoria, South Africa), there remains scant data on women in forensic mental health facilities, or those referred for psychiatric assessment under the *South African Criminal Procedure Act* (CPA) of 1977, and its amendments.^18,19^ Other published studies that have been previously conducted in South Africa on offenders referred by courts for psycho-legal evaluation under the CPA have either been confined to single sites, with relatively small sample sizes, or have focused primarily or exclusively on male offenders.^20,21,22,23,24,25^

## Aims

This cross-facility, national study examined the socio-demographic profile, court referral data and forensic mental health characteristics of accused women referred for psychiatric evaluation, under the CPA,^18,19^ to forensic mental health units in South Africa. The primary aim of the study was to document a detailed description of the psycho-social, clinical and forensic mental health profile of these women. In addition, the study also sought to focus particularly on women accused of violent offences, by exploring the possible motives, victim profiles and broader forensic mental health context of such violence.

## Methods

A retrospective study of clinical records of all accused women referred by the South African courts to all large forensic mental health units for observation under the CPA was conducted. The following institutions were included in the study: Fort England Hospital (Eastern Cape), Valkenberg and Lentegeur Hospitals (Western Cape), Weskoppies and Sterkfontein Hospitals (Gauteng), Fort Napier Hospital (Kwazulu-Natal), and the Free State Psychiatric Complex (Free State), respectively. Data were collected from the clinical forensic records of 573 women referred by courts for psycho-legal evaluation over a 12-year period (1998–2010), including information on psychiatric assessments, psychological tests, physical examinations, laboratory tests and clinical observations by multidisciplinary teams. Data were captured from clinical records, using a standardised data collection sheet across all participating sites. Specific information on the socio-demographic, criminological, clinical and forensic mental health profiles of the accused women was collected, collated and analysed. Descriptive analysis was computed on SPSS statistical software.

## Ethical consideration

Walter Sisulu University (Ref. No. 026/012); University of Cape Town (Ref. No. 278/2012); University of the Witwatersrand (Ref. No. R14/49); University of Pretoria (Ref. No. 109/2012); and University of Kwazulu-Natal (Ref. No. HRK M66/14). Formal ethics clearances from respective institutions were obtained from the managers of all participating forensic mental health units and through each affiliated University Human Research Ethics Committee, respectively. Investigators at each site signed a Declaration of Confidentiality and were responsible for ensuring the anonymity, confidentiality and security of data obtained at each site. Individual informed consent was not required, as this was a records-based study and permission to access records was obtained from the Head of Health Establishment at each institution. The views expressed in this article are those of the authors and do not represent the official position of any affiliated institutions or funders.

## Results

### Socio-demographics

The majority of the female offenders were aged between 21 and 50 years of age (82.0%), with the largest percentage being in the 21–30 years age group (33.2%). The majority of women (67.5%) were mothers, with almost half (46.2%) having more than one child, and almost 20.0% having more than three children. More than half of the sample were single at the time of observation (56.0%) and 3.7% were in common-law partnerships. A minority (13.6%) were divorced or separated. Regarding living arrangements prior to arrest: only 19.7% of the offenders lived with their partners or spouse, whilst 38.6% of the sample lived with their immediate family. Approximately 41.0% of the sample, whose income source was recorded, relied upon their immediate family to support them financially, with 78.0% of the sample, whose employment history was recorded, being unemployed prior to arrest. Despite this high rate of unemployment, only 23.0% were reliant on social grants for income, with the remaining unemployed offenders reliant on family, spouses and other sources. In terms of educational qualifications, only 6.1% of the sample were identified as having no formal education, with a further 4.4% of the sample with no educational level recorded. A high percentage of offenders had attained a Grade 8–12 education (49.0%), whilst only 8.5% had tertiary qualifications.

### Medical and psychiatric history

Women offenders who had a known prior medical history (43.7% of the sample) showed a disproportionately high rate of epilepsy (*n* = 64 or 11.2% of the whole sample, and 28.0% of those whose medical history was recorded). Only 2.8% (16/573) had evidence of prior traumatic brain injury, and 5.0% (29/573) were known to be HIV-positive. Forty-four per cent (*n* = 251) denied any prior substance abuse, whilst 38.3% (*n* = 220) of women disclosed and/or confirmed a history of alcohol abuse (i.e. reported this upon enquiry during clinical interviews and/or confirmed with collateral sources to this effect, respectively) during the course of the psycho-legal assessment. Alcohol was the most common with regard to substance abuse, accounting for almost 70.0% of those who disclosed prior substance abuse, followed by ‘other’ substances – used by less than 7.0% of the sample – including cannabis, heroin, nicotine, methamphetamine and prescription drugs. Substance abuse was a prevalent factor in offences committed against children, (*n* = 116 or 20%), with 32.0% of those who committed crimes against their own children reporting prior use of alcohol.

A prior psychiatric history was documented in almost half (48.9%; *n* = 280) of the women. In those whose previous psychiatric diagnosis was known, 12.2% were diagnosed with bipolar disorders, 11.9% with psychotic disorders and 10.8% with depressive disorders (Table 1). In addition, 25.0% (*n* = 141) of the women offenders had a documented history of mental illness in a family member.

**TABLE 1:** Psychiatric history prior to arrest (*n* = 573).

Psychiatric history	Frequency	%
Unknown/unspecified	293	51.1
Bipolar disorders	70	12.2
Psychotic disorders	68	11.9
Depressive disorders	62	10.8
Other mental disorders	46	8.0
Substance-related disorders	18	3.1
Anxiety disorders	7	1.2
Neurodevelopmental disorders	6	1.0
Personality disorders	2	0.3
Neurocognitive disorders	1	0.2

**Total**	**573**	**100.0**

Of those women who had been convicted of crimes against their own child or children (*n* = 116), 54.3% had disclosed a previous psychiatric illness. A closer analysis of this sub-sample reveals a relatively higher rate of depressive disorders (21.1%) and similar rates of psychotic disorders (11.2%) in comparison to the total sample.

### History of abuse experiences

Almost a third (30.8%) of the women offenders had disclosed a history of being abused. Of the 177 women who reported previous abuse experiences, 48.6% had a history of being abused as adults, 34.4% during childhood and 12.0% had experienced abuse both during childhood and adulthood (the remaining 5.0% reported abuse but the timing was unspecified). Of those in whom the type of childhood abuse was specified (65/573), 66.0% reported physical abuse, whilst 23.0% were sexually abused. For those who experienced abuse as adults, the most common type reported was physical abuse (75.0%). Sexual abuse was reported by 5.7% of these women, and 15.3% indicated they had been subjected to both sexual and physical abuse. A history of abuse was reported by almost one-third (28.6%) of women who committed violent offences.

### Criminal history

Historical offences were categorised using an adaptation of Snyman’s classification of offences for South Africa.^26^ There was no prior criminal history in over 60.0% of the sample (although a history of prior convictions was unknown or unspecified in a further 23.0%). Of those with documented prior convictions (*n* = 81), 60.0% were property offences (e.g. crimes relating to appropriation of property, financial crimes and damage to property). Almost 32.0% had prior convictions for violence against the person (e.g. murder, attempted murder, assault and assault with intent to do grievous bodily harm). Only 6.0% had previously committed ‘crimes against the community’, which includes a range of offences, many of them of a serious nature (e.g. crimes against the family, drug offences and concealment of birth). The most common convictions for specific prior offences committed were for assault (22.0%) and theft (23.0%), respectively, with only three cases of prior homicide.

### Offence profile

Index offences, which formed the basis of court-ordered referral for psycho-legal evaluation, were also categorised using an adaptation of Snyman’s classification,^26^ as follows: (1) *crimes against the State* (e.g. public violence, contempt of court, escape from custody); (2) *crimes against the community* (e.g. sexual crimes, abduction, corruption, drug offences, concealment of birth); (3) *crimes against a person*, which includes *crimes against life* (e.g. murder, attempted murder, culpable homicide, exposing an infant), *crimes against bodily integrity* (e.g. common assault, assault with intent to do grievous bodily harm, intimidation) and *crimes against dignity, reputation or freedom of movement* (e.g. defamation, kidnapping); and (4) *crimes against property* (e.g. theft, robbery, fraud, malicious injury to property, arson, housebreaking, trespass), respectively.

The most prevalent categories of offences were those against life (34.2%), property (30.2%) and bodily integrity (25.1%), respectively. The most prevalent specific index offences are set out in Table 2.

**TABLE 2:** Five most prevalent index offences.

Type of offence	Frequency	%
1. Murder	169	29.5
2. Assault with intent to do grievous bodily harm	95	16.6
3. Theft	73	12.7
4. Fraud	29	5.1
5. Attempted murder	26	4.5

In total, 55 (68.0%) of those with previous convictions were accused of violent index offences. Of those who had committed non-violent crimes on first offence, two-thirds went on to commit violent crimes; for example, 35 out of the 81 women with prior convictions went on to commit a murder or attempted murder.

### Apparent motives of offence

The motives for committing the offence against adult victims were recorded as being unknown/unspecified in 67.7% of the sample (*n* = 388). Of those in whom motives were specified (*n* = 185), psychopathology was cited as a significant factor in driving behaviour in 39.4% (*n* = 73), 37.2% (*n* = 69) committed the offence as a form of retaliation/revenge, 13.0% (*n* = 24) of the offences were motivated by self-defence and the remaining 10.4% (*n* = 19) were recorded as having ‘other’ motives (e.g. for financial gain, intoxication or sexual exploitation). With regard to those crimes which were motivated by psychopathology (*n* = 73), the prevailing mental state during the offence was recorded as being psychotic in 29.0% (*n* = 21) of the cases, with a further 10.8% experiencing a manic or major depressive episode at the time.

With regard to crimes committed against children, the likely motives were classified using an adaptation of the typology of D’Orban^27^ and, as illustrated in Table 3, active psychopathology at the time of offending accounted for the majority of cases (33.6%).

**TABLE 3:** Motive for offences against offenders’ own child (*n* = 116).

Motive	Frequency	%
Psychopathology of accused	39	33.6
Unknown^a^	37	31.9
Unwanted child	14	12.1
Not recorded^b^	10	8.6
Retaliation/revenge	7	6.0
Impulsive	5	4.3
Other^c^	4	3.4

**Total**	**116**	**100.0**

a , ‘Unknown’ refers to cases in which the motive was truly unknown to the clinical team;

b , ‘Not recorded’ refers to cases in which clinical records information did not document the motive;

c , ‘Other’ refers to recorded motives which did not fit any other category.

Similar to the finding with adult victims, the most common form of psychopathology at the time of offending was psychosis (29.7%), with a further 8.5% experiencing a manic or major depressive episode. Of note, a large proportion (32.6%) were considered to have normal mental states (i.e. devoid of active psychopathology) during the commission of offences against children.

### Victim profiles

#### Adult victims

A total of 398 adult victims were reported across the sample (although in some cases, a single offender targeted more than one victim). Of these, 27.3% (*n* = 109) were in respect of crimes against life (e.g. murder, attempted murder), 29.0% (*n* = 115) were property-related crimes and 16.5% (*n* = 66) were categorised as crimes against bodily integrity (e.g. assault and assault with intent to do grievous bodily harm). More specifically, 94 out of the 398 crimes against adults were murders, which equates to 23.6% of the crimes of the whole sample (*n* = 573). Of those offenders who committed crimes against adults, only 58.0% (*n* = 230) of the records specified the offenders’ relationship with the adult victim. Of those relationships which were identified in the records: 29.0% (*n* = 66) of the victims were acquaintances or friends of the accused, 22.0% (*n* = 50) were relatives, 18.6% (*n* = 43) were intimate partners, 14.3% parents and 16.0% were strangers to the offender. Overall, of all the violent crimes (*n* = 360) that were committed, 9.2% were perpetrated against a partner, with a combined total of 31.7% of all violent crimes targeting adult victims known to the offender (including partners, family members, acquaintances and friends).

#### Child victims

In total, there were 175 crimes committed against children, with 66.3% (*n* = 116) of victims being the biological children of perpetrators. The age of the child victim was recorded in 129 (74.0%) of all cases, as illustrated in Table 4. Almost a third of all child victims (31.0%; *n* = 54) were aged under 1 year at the time when the crime was committed against them. For cases of offenders committing crimes against their *own* children (*n* = 116), children aged 1 year were frequently targeted (38%; *n* = 44), with the first month of life being a particularly vulnerable age (18.0%; *n* = 21).

**TABLE 4:** Age distribution of child victims (*n* = 175).

Age category	Biological child victims		All child victims	
	Frequency	%	Frequency	%
0–1 month old	21	18.1	28	16.0
2–11 months	23	19.8	26	14.9
1–4 years	21	18.1	34	19.4
5–8 years	13	11.2	23	13.1
9–12 years	5	4.3	11	6.3
13–16 years	3	2.6	7	4.0
Unknown/unspecified	30	25.9	46	26.3

**Total**	**116**	**100.0**	**175**	**100.0**

Of the 175 child victims in whom the gender was specified/known, 29.1% were female (*n* = 51), 18.6% were male (*n* = 33), with 4.6% of the crimes committed against multiple victims of both sexes (*n* = 8). Regarding the gender of biological child victims in whom this was specified/known (*n* = 116), 24.0% were female, 19.0% were male and 3.4% were of both sexes.

Where cases involved violent offences against children, the majority (69.8%) were against biological children (*n* = 81 out of 116 cases were violent crimes). With regard to the offences against life against their *own* children: 34.4% of these crimes were murders (accounting for 24.0% of all murders [*n* = 40 out of 169] in the whole sample) and 4.3% (*n* = 5) of these were attempted murder (17.0% of all attempted murders within the whole sample). Important factors in relation to child deaths relate to the history of psychiatric illness in the female offender’s family and her own previous psychiatric history. Of those 175 women who committed crimes against children, 6.2% (*n* = 11) identified their mothers as having had a previous history of psychiatric illness. Out of those 11 women, four had murdered or attempted to murder a child. Regarding the offenders’ own history of psychiatric illnesses, 52.0% (*n* = 90 out of the 175 women) had disclosed a prior psychiatric illness, and of these 90 women, 30.0% had murdered a child.

### Psycho-legal referral

The most common reasons for being referred by courts for psychiatric observation include clinically related referrals in 45.0% of cases (e.g. prior psychiatric or neuropsychiatric history, or upon recommendation of a health professional) and criminal justice referrals comprised 28.0% of the sample (e.g. unusual offence characteristics, difficulties with lawyers consulting with accused women and unusual behaviour in court). The remainder were for other, unspecified or unknown reasons.

### Diagnostic outcomes

The final diagnostic outcomes following psycho-legal evaluation are illustrated in Table 5. Almost one-third (29.0%; *n* = 164) had no mental disorder. With respect to violent crimes in particular (*n* = 360): 21.9% were diagnosed with a psychotic disorder, 9.7% with bipolar disorder and 5.3% with a depressive disorder.

**TABLE 5:** Diagnostic groups and the nature of offences (*n* = 565).^a^

Diagnostic group^b^	Violent and non-violent crimes				Total	
	Violent		Non-violent			
	*N*	%	*N*	%	*N*	%
No mental disorder	108	30.0	56	27.3	164	29.0
Schizophrenia and other psychotic disorders	79	21.9	48	23.4	127	22.4
Comorbidity	34	9.4	35	17.1	69	12.2
Bipolar and related disorders	35	9.7	21	10.2	56	9.9
Other mental disorders	20	5.5	7	3.4	27	4.8
Personality disorders	19	5.3	9	4.4	28	5.0
Depressive disorders	19	5.3	8	3.9	27	4.8
Substance-related and addictive disorders	12	3.3	10	4.9	22	3.9
Neurocognitive disorders	16	4.4	2	1.0	18	3.2
Neurodevelopmental disorders	11	3.1	6	2.9	17	3.0
Trauma and stressor-related disorders	5	1.4	3	1.5	8	1.4
Anxiety disorders	2	0.6	0	0.0	2	0.4

**Total**	**360**	**100.0**	**205**	**100.0**	**565**	**100.0**

a , The eight ‘Unknown’ cases have been excluded from the table and the calculations above;

b , Diagnostic grouping aligned with DSM-5 (diagnostic and statistical manual); the ‘Comorbidity’ group includes cases with two or more discrete psychiatric diagnoses made on court reports following psycho-legal assessment; the ‘Other Mental Disorders’ group includes cases with diagnoses in categories other than the Diagnostic Groups indicated.

Of the 127 offenders with a diagnosed psychotic disorder, 26.0% had committed murder or attempted murder, and 32.8% had committed common assault or assault with intent to cause grievous bodily harm. Interestingly, whilst 48.0% of those with depressive disorders had also committed a high number of crimes against life (i.e. murder or attempted murder) (13 out of the 27 offenders), this is notably lower than the absolute number of offenders diagnosed as having no mental disorder, of whom 73 of 166 women had also committed murder or attempted murder.

### Trial competence and criminal responsibility

Conclusions on both trial competence and criminal responsibility were specified in only 511 reports. Table 6 illustrates that the majority of women were deemed to be trial competent (61.4%; *n* = 314) and criminally responsible for the crimes (52.2%; *n* = 267), with over half the female offenders reported as *both* trial competent and criminally responsible (50.8%; *n* = 260). Of the female offenders who committed violent crimes (*n* = 360), 61.7% were declared trial competent. Of those who committed crimes against children, 58.9% were deemed trial competent. This increased slightly to 61.2% amongst those women who committed crimes against their own children (*n* = 116), with full criminal responsibility present in 45.7% of cases.

**TABLE 6:** Trial competence and criminal responsibility (*n* = 511).

Criminal responsibility	Trial competence					
	Fit		Not fit		Total	
	*N*	%	*N*	%	*N*	%
Responsible	260	82.8	7	3.6	267	52.2
Not responsible	54	17.2	190	96.4	244	47.8

**Total**	**314**	**100.0**	**197**	**100.0**	**511**	**100.0**

Final recommendations to court were specified in 502 court reports. In the majority (61.0%) of cases, this was for the law to take its course. The remainder were recommended for referral to mental health services for clinical treatment and/or rehabilitation: to general mental health services in 18.1% (*n* = 91) in cases of non-violent index offences; and forensic mental health services in 20.9% (*n* = 105) in cases of violent index offences, respectively.

## Discussion

The clinical records of 573 women offenders referred by courts for psycho-legal evaluation under the CPA to the six South African participating forensic mental health units were examined.

### Pre-arrest profile

From this study, the ‘typical’ female offender is a single mother, aged between 21 and 30 years, with one or more dependent children. She would have attained a Grade 8–12 school education, would be unemployed prior to the offence and living with her immediate family, upon whom she would be reliant for financial and other forms of support. Almost a third of women disclosed a pre-arrest history of having experienced abuse themselves, both during childhood and adulthood, in keeping with the body of evidence that women are more likely than male offenders to report extensive histories of physical, sexual and emotional abuse.^28^ Rivera and Widom demonstrated that any type of childhood abuse serves as a predictor for being arrested for a violent crime during adulthood.^29^ The predictive element of early trauma is not only connected to criminal behaviour but also to the subsequent development of mental disorders, including substance-related problems. It is unsurprising then that the majority of women in the sample had a documented psychiatric history, most commonly suffering from mood or psychotic disorders, and that almost 40% of the women disclosed a prior history of alcohol abuse. Most of the women sampled were first-time offenders with no prior criminal history. In those with prior convictions, the majority were for minor, non-violent property-related offences, whilst a small but notable proportion had prior violent offending histories. This pre-arrest profile, and the associated pathways to offending, is congruent with that described in the international literature.^2,6,10,12,13,14^

### Index offence profile

The most prevalent offences that led to court-referred forensic psychiatric evaluations were for violent index offence categories: offences against life (e.g. attempted murder and murder) and against bodily integrity (e.g. assault and assault with intention to do grievous bodily harm). In fact, murder was the single most common index offence. Women who were first-time offenders were more likely to commit non-violent index offences, and those with a prior criminal history (of any kind) were more likely to engage in violent index offences. Psychopathological factors were also cited as a significant factor associated with violent index offending, cited in over one-third of the cases. This relatively large proportion of violent offending (historical and related to the index offence) in South African female offenders, especially in the context of mental health problems, confirms the conclusions of other published research abroad.^12,13,14,30,31^

Overall, the study demonstrated that victims of violent offending were predominantly situated within the women’s immediate family and their direct inner social circle. Adult victims of violent offences were known to the offender in over 80.0% of cases, as a family member, intimate partner, friend or acquaintance. Intimate partners were the single largest group of victims of homicidal offences, which includes murder and attempted murder (comprising 25.0% of cases, as compared with only 8.0% of such cases being unknown to the perpetrator), confirming the evidence in the literature on violent female offenders and their victims, especially in the context of intimate partner violence (IPV). As an example, a large Swedish study of the court judgements of women who killed their intimate partners by Moen, Nygren and Edin^32^ reported that the homicidal act often occurred on the background of prior violent abuse of the women themselves at the hands of their victims. Female-perpetrated IPV has been linked to defensive reactions related to chronic prior abuse.^33^ South African women, like women elsewhere, are more likely to be victims than perpetrators of IPV: it is estimated that at least 31.0% – 55.0% of women in South Africa have experienced IPV.^34^ Breet, Seedat and Kagee^35^ articulate that poor mental health is a crucial risk factor for the perpetration of IPV in South Africa and recommended that IPV can only be tackled if gender-sensitive interventions consider co-occurring symptoms of mental ill-health, especially depressive and anxiety syndromes.^35^

Outcomes with respect to child victims in the current study also confirm the patterns described in the literature: biological children in their first year of life are a particularly vulnerable group, especially in cases where maternal perpetrators are likely to be suffering from mental ill-health.^36,37,38^

### Forensic mental health profile

The fact that severe mental disorder was present in a relatively high proportion of cases suggest that mental ill-health confers a disproportionate risk of offending in women in our sample (especially violent offending in the presence of psychotic-spectrum disorders), a conclusion consistent with those of most other large studies in the published literature.^9^ Despite the presence of a psychiatric diagnosis in two-thirds of women offenders, approximately half (51.0%) were deemed to be trial competent and criminally responsible for their actions. The psychiatric disorder may have been in remission at the time of offence (in relation to criminal responsibility) and/or following arrest, that is, at the time of forensic psychiatric evaluation itself (in relation to trial competence). Another possibility is that, even if these disorders were not in remission at the time of trial or offence, the symptoms of the disorder did not sufficiently impact on the forensic parameters of trial competence and/or criminal responsibility as to deflect the law from taking its course in the usual manner.

## Conclusion

There are a number of limitations to this research study, including the retrospective nature of the study, site-differences in the quality of clinical records documentation and information to hand, and the lack of male offender comparative data. There are further limitations regarding the generalisability of results and outcomes to women in other settings or populations, for example, women in the community, or convicted women serving sentences in prison. Nonetheless, this is the largest and most comprehensive survey of the clinical and forensic mental health of women offenders in South Africa (and, to our knowledge, on the African continent as a whole) to date, and a number of broad conclusions can be drawn. Most women offenders sampled come from a background of socio-demographic and socio-economic adversity, with a relatively high pre-offence incidence of mental ill-health, alcohol abuse and being victims of abuse themselves. Whilst the majority of women were first-time offenders, the majority of index offences which led to their court-ordered forensic evaluation were for violent offences against the person, with murder being the single most common index offence. Most victims of such violence were known to the perpetrators as family members, intimate partners or biological children. There were relatively high rates of psychotic and mood-spectrum disorders present in the sample, and relatively low rates of personality disorders. The majority of women were deemed to be trial competent and criminally responsible in relation to their index offences. Further research, especially in the context of developing countries, is necessary to confirm the outcomes of this study, and to expand sampling to male offenders in order to generate gender-based comparisons. In addition, it is recommended that more standardised and gender-sensitive forensic mental health assessment approaches, documentation and reporting be employed throughout the country.
